# Mitigating RNA Toxicity in Myotonic Dystrophy using Small Molecules

**DOI:** 10.3390/ijms20164017

**Published:** 2019-08-17

**Authors:** Kaalak Reddy, Jana R. Jenquin, John D. Cleary, J. Andrew Berglund

**Affiliations:** 1 The RNA Institute, University at Albany–SUNY, Albany, NY 12222, USA; 2 Center for NeuroGenetics and Biochemistry & Molecular Biology, University of Florida, Gainesville, FL 32608, USA

**Keywords:** myotonic dystrophy, small molecules, therapies

## Abstract

This review, one in a series on myotonic dystrophy (DM), is focused on the development and potential use of small molecules as therapeutics for DM. The complex mechanisms and pathogenesis of DM are covered in the associated reviews. Here, we examine the various small molecule approaches taken to target the DNA, RNA, and proteins that contribute to disease onset and progression in myotonic dystrophy type 1 (DM1) and 2 (DM2).

## 1. Introduction

**Figure 1:**
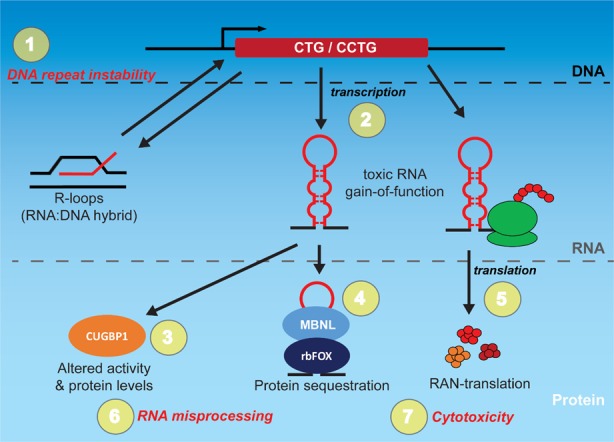
Multiple levels of disease mechanism and therapeutic targets in myotonic dystrophy (DM). A simplified schematic with both RNA gain-of-function (GOF) and repeat-associated non-ATG (RAN) translation protein products is illustrated. The specific proteins and processes vary between sense and antisense transcripts and between myotonic dystrophy type 1 (DM1) and 2 (DM2), with a generic process shown for review processes. Repeat expansion in the DNA is the source of numerous downstream pathogenic processes. Bi-directional transcription of CTG and CCTG repeat expansions produce toxic sense (CUG/CCUG) and antisense (CAG/CAGG) expansion RNA that can fold into hairpin structures with pathogenic downstream consequences. Co-transcriptional R-loops (a type of RNA:DNA hybrid) can trigger DNA repeat instability [1,2,3] which is positively correlated with increased disease severity. Toxic expansion RNAs can sequester important proteins, such as the MBNL and rbFOX family members [4], and trigger hyperphosphorylation of CUGBP1 leading to its increased steady state levels and altered activity. Toxic sense and antisense RNA can also trigger repeat-associated non-ATG (RAN) translation in multiple reading frames, producing toxic aggregating peptides that lead to cytotoxicity. Each of these different stages (expansion DNA, RNA, and protein) and their associated defects (**1–7**) represent a potential target of small molecules with unique advantages and disadvantages. Targeting the DNA repeat expansion (**1**) using small molecules for deletion or contraction should theoretically eliminate all downstream pathogenic process. While this type of treatment could also involve a single or few doses to achieve a permanent effect, it is technically challenging and there is little current progress on this front. Compounds that target transcription (**2**) may also alleviate many of the downstream consequences at lower doses compared with approaches that target the toxic RNA molecules itself, but will require continuous administration. Most progress in small molecule therapeutics for DM to date has been from targeting specific downstream consequences (**3–7**). While there are multiple targets, this approach is the furthest developed with several compounds showing considerable efficacy and progress in clinical trials. For example, there is a current phase II clinical trial with Tideglusib that modulates CUGBP1 activity.

The development of therapeutic approaches based on small molecules offer several advantages over other therapeutic strategies. Small molecules can be administered orally, and generally have better tissue delivery with shorter half-lives and longer shelf lives than other biologics, making rapid reversal of treatment easy in case of toxicity. Small molecules typically have lower costs associated with manufacturing, offer opportunities for repurposing, and most notably, are amenable to high-throughput screening and optimization using medicinal chemistry-based approaches. Excitingly, there are a number of small molecules being used to target the different aspects of the ‘toxic RNA cycle’ in myotonic dystrophy (DM) (Figure 1) to restore normal cell function (Tables S1–S5).

Since the discovery of the toxic RNA gain-of-function mechanism in myotonic dystrophy type 1 (DM1) and 2 (DM2) [5], various small molecule strategies have been employed to target the toxic sense (CUG/CCUG) expansions for therapeutic benefit (see other recent reviews from Artero and Brooks’ labs [6,7]). DM thus serves as an important paradigm for small molecule-mediated targeting of RNA as a therapeutic design. Initial studies were focused on displacing or preventing muscleblind-like (MBNL) protein binding to the CUG/CCUG expansion RNA. More recently, sophisticated compounds have been designed that can specifically target toxic RNA for degradation. This evolution in small molecule studies in DM stemmed from an understanding of the CUG/CCUG repeat expansion RNAs and the secondary structures they adopt as the toxic entities of pathogenesis. 

Structural studies of CUG/CCUG repeats provide valuable clues for the screening, selection, and rational design of lead compounds that may selectively interact with CUG/CCUG RNA and displace sequestered MBNL proteins or block their sequestration. Early in vitro structural studies of r(CUG)_n_ sequences using UV melting and nuclease/lead ion structure probing experiments identified the propensity of CUG RNA repeats to fold into stem-loop hairpin structures in a repeat length-dependent manner [8,9]. High-resolution X-ray crystallography structures have been solved for both r(CUG)_n_ repeats and r(CCUG)_n_ repeats, revealing fine detail of both the helical shape and loop dynamics of the hairpin structures [10,11]. For more detailed information, see a recent review on the structures of CUG/CCUG repeats and other disease-causing repeats published by Kiliszek and colleagues [12]. These structural studies provide useful information in understanding how small molecules may potentially interact with their CUG/CCUG expansion targets. 

## 2. Targeting the Toxic RNA in DM through Rational Design

### 2.1. Triaminotriazine-Based Designs

Based on the published CUG hairpin crystal structure [10], the weakly-interacting U-U mismatches within the hairpin were hypothesized to present a targetable site for which a small molecule could be developed to interact through hydrogen bonding [13]. Ligand 1 was developed by conjugating an acridine unit for DNA intercalation and a triaminotriazine unit for Janus-wedge-type hydrogen bonding to the U-U mismatch [10] (Table S1). This compound was effective at binding to both CTG and CUG oligo sequences in the high nanomolar range, preferentially recognizing T-T and U-U mismatches over C-C, A-A, and G-G mismatches and duplex substrates [13]. In a test of its efficacy for treatment of DM, ligand 1 was shown to effectively disrupt MBNL1:r(CUG)_4_ and r(CUG)_12_ complexes in vitro in the mid-micromolar range [13]. While effective in disrupting MBNL1:r(CUG)n complexes, ligand 1 demonstrated low solubility, low cell penetration, and high cell toxicity, leading the authors to pursue alternative compounds. 

The second generation of triaminotriazine compounds developed included ligand 3 (Table S1), which contains two triaminotriazine groups separated by a bisamidinium linker permitting targeting of three consecutive CUG units [14]. Ligand 3 effectively bound r(CUG)_12_ and disrupted MBNL1:r(CUG)_12_ complexes in vitro in the low micromolar range, offering a significant improvement over the earlier generation ligand 1. The efficacy of ligand 3 in rescuing the molecular phenotypes of DM was tested by treating HeLa cells transiently expressing r(CUG)_960_, which reduced ribonuclear foci and partially reversed MBNL-dependent mis-splicing of cardiac troponin T (*cTNT*) exon 5 and insulin receptor (*INSR*) exon 11 in the mid-micromolar range [14]. Additionally, treating a *Drosophila* DM1 transgenic model expressing r(CUG)_480_ interrupted repeats with ligand 3 rescued a glossy eye phenotype relating to CUG RNA toxicity in the high micromolar range [14]. The low cytotoxicity and high solubility of ligand 3, coupled with its activity in cell culture and *Drosophila* models, makes this small molecule an improved candidate therapeutic molecule for DM1. 

In an effort to expand target engagement in DM1 by individual compounds, new triaminotriazine-based ligands were developed with added ammonium groups and imidazole or amino groups. These added catalytically active groups allowed for the targeting of CTG DNA, CUG RNA, and had cleavage capability of CUG RNA similar to that of an RNase A enzyme [15]. The most promising of these compounds, ligand 9 (Table S1) was able to block T7 in vitro transcription from plasmids containing a (CTG)_74_ tract and selectively cleave an r(CUG)_16_ oligo substrate in vitro. In cell culture, treatment with ligand 9 disrupted ribonuclear foci, reduced r(CUG)_EXP_ transcript levels, and rescued *INSR* exon 11 minigene splicing [15] in HeLa cells transfected with an interrupted (CTG)_960_ plasmid. Similar to ligand 3, ligand 9 reversed the glossy eye phenotype in DM1 transgenic *Drosophila* and also rescued larval locomotor defects associated with CUG RNA toxicity while reducing r(CUG)_EXP_ transcript levels [15]. The improved performance of this compound relative to previous triaminotriazine ring-based ligands speaks to the power of rationale and incremental drug design.

In a recent refinement of the triaminotriazine-based compounds, a new oligomeric compound was reported—ligand 4 (Table S1), composed of multiple alternating bisamidinium units and triaminotriazine groups [16]. This oligomeric mixture, composed of 4–8-mers, displayed positive binding cooperativity of an r(CUG)_16_ substrate in vitro and selectively inhibited transcription of a CTG expansion in vitro [16]. Ligand 4 was able to reduce r(CUG)_EXP_ levels in HeLa (CTG)_960_ transfection experiments in the high nanomolar to low micromolar range [16] and reduce ribonuclear foci in DM1 patient-derived myoblasts. While splicing correction in the latter cell line was not reported, the compound did display positive effects in vivo in both a *Drosophila* and DM1 liver mouse model [16]. Treatment of DM1 transgenic flies expressing r(CUG)_480_ with 20–80 micromolar of ligand 4 rescued a progressive climbing defect associated with neuromuscular dysfunction [16]. In a novel DM1 liver-specific inducible mouse model producing r(CUG)_960_, intraperitoneal (ip) injection of ligand 4 into the liver reduced MBNL1-containing ribonuclear foci and rescued several MBNL-dependent mis-splicing events [16]. These studies support the feasibility of multivalent approaches to target various stages of the toxic RNA process in DM. However, it will be important to determine the permeability and bioavailability of such compounds in other relevant tissues such as skeletal muscle, which remains a challenge in small molecule administration.

### 2.2. 2,9-Diaminoalkyl-1,10-phenanthroline (DAP)-Based Designs—An Independent Rationale Design Approach 

An independent effort exploiting the structure of CUG repeats to drive ligand design, targeted hydrogen bonding with the U residues of the CUG hairpin. This approach lead to the development of a compound termed DAP (2,9-diaminoalkyl-1,10-phenanthroline) (Table S1), which was capable of binding to the CUG RNA [17]. Molecular modelling simulations supported DAP interaction with the CUG RNA through hydrogen bonding to an intrahelical uracil residue of the U-U mismatch and interacting with adjacent guanines of the C-G base pair through π–π stacking [17]. Initial characterization of DAP using surface plasmon resonance (SPR) and UV melting experiments supported its selectivity to r(CUG)_9_ RNA substrates compared to r(CAG)_9_ and r(CGG)_9_. Interestingly, DAP had comparable affinity for r(CCG)_9_ RNA [17] and also bound to d(CTG)_9_ and d(CCG)_9_ substrates [17]. While these initial studies demonstrated a sub-optimal specificity of DAP for CUG RNA, a more recent refinement of the DAP compound has sought to improve specificity. This approach relied upon multivalence to increase selectivity by synthesizing a DAP dimer (DDAP) where the two DAP monomers were linked through an amide group (Table S1) [18]. The improved DDAP compound exhibited increased affinity for both r(CUG)_9_ and r(CCG)_9_ substrates relative to DAP [18]. To assess the potential of DDAP as a therapeutic for DM1, a mouse C2C12 myoblast transgenic cell model that expresses r(CUG)_800_ was treated with DDAP, and the reversal of the mis-splicing of the sarcoplasmic/endoplasmic reticulum calcium-ATPase (Atp2a1 or Serca1) exon 22 was monitored [18]. DDAP treatment induced the reversal of mis-splicing at 40 µM in this system without significant cytotoxicity. Treatment of the DM1 mouse model expressing approximately 220 CTG repeats in the human skeletal actin gene (HSA^LR^) with DDAP through ip injection partially rescued the mis-splicing of the chloride channel (Clcn1) exon 7a and Atp2a1 exon 22 [18]. Filter binding assays confirmed that DDAP inhibited the interaction of the MBNL1 protein to r(CUG)_20_ RNA in the nanomolar range in vitro, supporting the predicted mechanism of action [18]. The fact that DAP induced considerably higher cytotoxicity than DDAP at equal concentrations supports the favourable effect of multivalence to improve targeting of the r(CUG)_EXP_ while reducing off-target binding [18]. It will be interesting to determine if further oligomerization of DAP monomers yield additional compounds exhibiting increased selectivity for r(CUG)_EXP_ RNA and favourable bioactivity. 

### 2.3. Kanamycin and other Derivatives to Target the DM2 CCUG RNA

Initial small molecule studies focused on targeting the r(CUG)_EXP_ driving pathogenesis in DM1. However, identification of high-affinity pyrimidine-rich 2 × 2 internal loop recognition by 6′-*N*-5-hexynoate kanamycin A, a kanamycin derivative, facilitated the design of compounds targeting the toxic CCUG RNA of DM2 [19,20,21]. Several ligands developed through modular assembly, starting with 6′-*N*-5-hexynoate kanamycin A, yielded compounds with low nanomolar affinity greater than that of MBNL1 for CCUG RNA in vitro [21]. Importantly, these compounds were able to enter mouse C2C12 myoblasts, including limited entry into the nucleus. In subsequent work, derivatives containing multiple 6′-*N*-5-hexynoate kanamycin A modules with optimized propylamine spacers to facilitate r(CCUG)_EXP_ binding were assessed in C2C12 cells co-transfected with plasmid expressing r(CCUG)_300_ and a *BIN1* exon 11 mini-gene. The parent compound 6′-*N*-5-hexynoate kanamycin A, and derivatives 2K-4 and 3K-4 (Table S1), significantly rescued mis-splicing in the low to mid micromolar range [11]. Notably, because of the similar structure of r(CUG)_EXP_ and r(CCUG)_EXP_ RNA, varying the spacer modules of the same 6′-*N*-5-hexynoate kanamycin A derivatives yields compounds with greater affinity for the r(CUG)_EXP_, highlighting the potential for modulating both toxic RNAs in DM using the same or similar scaffolds with modified linkers and spacers [22].

In a similar manner, the Zimmerman lab identified compounds structurally related to ligand 1 (showing affinity for CUG RNA) that have high affinity for CCUG RNA and inhibit MBNL1 binding to CCUG in vitro [23,24]. The addition of a triaminopyrimidine module facilitated recognition of C-U mismatches formed by CCUG RNA secondary structures. Thus, new CCUG ligands 6, 8, 10, and 11 (Table S1) synthesized with triaminopyrimidine units, and bisamidinium groove binding modules inhibited the MBNL1:CCUG RNA interaction in vitro in the low micromolar range. These compounds were also able to disrupt MBNL1:CCUG ribonuclear foci in HeLa cells co-transfected with (CCTG)_1200_ and GFP-MBNL1 plasmids at a treatment dose of 100 micromolar [24]. Importantly, these ligands displayed a favorable toxicity profile in HeLa cells at the treatment dose range. Thus, understanding ligand affinity for either CUG or CCUG RNA can inform the targeting of both toxic RNA, an important factor in the rationale design of therapeutic small molecules. 

### 2.4. The Development of Cugamycin

The wealth of existing knowledge can provide a starting point for the design of small molecules. By researching annotated RNA:ligand interactions and mining literature on nucleic acid interacting modules, the Disney lab identified *bis*-benzimidazole (Table S1) as a high affinity ligand for the U-U internal loop present within the r(CUG)_EXP_ [25,26,27]. Modular assembly of multiple *bis*-benzimidazole units on a peptoid backbone yielded a series of multivalent compounds capable of binding CUG RNA and inhibiting the MBNL1:r(CUG)_109_ interaction in vitro in the nanomolar range [26]. Dimeric compound 2H-4 (Table S1) was found to be the most bioactive and potent molecule in DM1 cellular assays [28]. 2H-4 treatment of HeLa cells transfected with an interrupted (CTG)_960_ plasmid and a *cTNT* exon 5 mini-gene reduced ribonuclear foci and reversed mis-splicing in the mid micromolar range [28]. To improve upon the bioactivity of 2H-4, 2H-K4NMeS was developed as a *bis*-benzimidazole dimer on a *N*-methyl peptide backbone capable of recognizing two sequential U-U loops in CUG RNA (Table S1) [29,30]. Treatment of DM1 patient-derived cells with 2H-K4NMeS, reversed MBNL1-dependent mis-splicing of several pre-mRNA, including *MBNL1* exon 5, and partially reduced the number of CUG ribonuclear foci [30]. While phenotypic rescue is critical, understanding compound selectivity within cells is another critical selection criterion. By modifying 2H-K4NMeS to contain chlorambucil and biotin for cross-linking and purification, respectively, Chem-CLIP was employed to assess RNA selectivity of the compound in cells [30]. Treatment and analysis of DM1 and control patient cells in this manner revealed a reported enrichment of *DMPK* mRNA of ~13,000 fold without any observed enrichment in non-DM1 cell lines, supporting the specificity of the compound in a cellular context [30]. While other RNAs were not found to be enriched within two orders of magnitude relative to *DMPK* RNA, the additive effects of “mild” off-target engagement in the context of an organism may be significant, depending upon the nature of the target. Therefore, for 2H-K4NMeS and any small molecule therapeutics, it is critical to relate low off-target levels to the overall health of an organism. 

Based on the selectivity for the CUG expansion afforded by 2H-K4NMeS, further modifications to the compound were explored to generate additional applications [30]. Conjugation of bleomycin, a natural peptide compound with RNA-cleaving capability, to 2H-K4NMeS generated a compound capable of selectively degrading r(CUG)_EXP_ RNA which was named Cugamycin by the Disney group [30,31]. Assessment of Cugamycin in DM1 patient-derived myotubes and the *HSA*^LR^ mouse model yielded favorable results in a number of pathogenic hallmarks, including the reduction of ribonuclear foci, rescue of MBNL-dependent mis-splicing, and partial reversal of myotonia [31]. This compound, which has received considerable recent attention, highlights the potential of logic-driven rational design and scaffolding to generate small molecules with multiple applications and improved therapeutic potential. 

## 3. Screening Small Molecule Libraries to Target Toxic CUG RNA

### 3.1. Combinatorial Chemistry Screen

Screening small molecule libraries is an important first step in the development of lead compounds for disease therapeutics. For targeting toxic CUG RNAs, one of the initial screening approaches to identify CUG-binding compounds used resin-bound dynamic combinatorial chemistry (RBDCC). This process relies on the sulfide exchange of amino acids and carboxylic acids in solution with resin-immobilized monomer scaffolds to assemble large numbers of peptide compounds [32,33]. Using this approach, an in vitro fluorescent CUG-binding screen of potentially over 11,000 peptide molecules identified several compounds displaying high-affinity binding to CUG RNA. These compounds were also able to inhibit MBNL1 binding to an r(CUG)_109_ expansion substrate in vitro in the low micromolar range [33]. Additionally, two of these compounds (4 and 11, Table S2) displayed partial rescue of MBNL-dependent mis-splicing in the *HSA*^LR^ mouse model. This effect was modest likely owing to size, stability, and bioavailability limitations of the compounds (Table S2) [34]. Since screening compounds in this manner is unlikely to yield an ideal candidate from the screen, additional refinement medicinal chemistry is essential to yield more potent and bioactive compounds. 

### 3.2. The Identification of Diamidines from Screens of Nucleic Acid Binding Molecules

Another early screen tested the activity of a small collection of known nucleic acid binders to disrupt purified MBNL1:r(CUG)_4_ complexes in an in vitro electrophoretic mobility shift assay. This approach identified pentamidine (Table S2), an FDA-approved anti-microbial drug, as a lead compound [35]. Pentamidine is currently used to treat trypanosomiasis and leishmaniasis parasitic infections, as well as pneumonia (*Pneumocystis carinii*), in immunocompromised individuals [36]. Pentamidine has been shown to interact with RNA to disrupt group I intron splicing and translation, leading to reduced cell growth in fungi [37,38,39]. Furthermore, based on interactions with DNA, pentamidine is also thought to inhibit kinetoplast DNA replication, as has been observed of related aromatic diamidine compounds in trypanosomes [36,40,41]. In relation to DM1, pentamidine treatment of a HeLa DM1 cell model transiently expressing r(CUG)_960_ revealed a reduction in MBNL:r(CUG)_EXP_ foci and the rescue of *INSR* exon 11 and cardiac troponin T (*cTNT*) exon 5 MBNL-dependent minigene mis-splicing events [35]. Treatment of the *HSA*^LR^ mouse model partially rescued MBNL-dependent mis-splicing of *Clcn1* exon 7a and *Atp2a1* exon 22. However, substantial toxicity was observed in treated mice, suggesting further work would be required to balance the trade-off between rescue and toxicity. Additional work on pentamidine’s mechanism of action demonstrated that it may be active at both the RNA and DNA levels with an insufficient specificity that likely contributes to the observed toxicity [42]. Overall, the strong rescue of mis-splicing made pentamidine an excellent lead nucleic acid binder candidate for further refinement. 

To identify pentamidine derivatives with improved activity, a structure activity relationship (SAR) study was undertaken. Increasing the methylene linker length in the compound between three to nine carbons correlated with increased efficiency of mis-splicing rescue in a DM1 HeLa minigene reporter cell model [42]. However, increased linker length also correlated with decreased solubility and increased toxicity. Heptamidine (Table S2), a seven-carbon linker derivative, displayed the highest efficacy of mis-splicing rescue while retaining water-solubility [42]. Heptamidine also rescued mis-splicing and myotonia in the *HSA*^LR^ DM1 mouse model and selectively reduced the long repeat *HSA* transgene mRNA levels versus short repeats. Heptamidine also demonstrated high toxicity, as some mice were not able to tolerate treatment doses in which pentamidine was not toxic. Given the promising rescue of diamidines, further refinement of the mechanism of action coupled with structure activity relationship experiments was performed. The approach led to the identification of furamidine (Table S2), which is capable of binding to CUG RNA, disrupting MBNL1 binding, reducing ribonuclear foci, and rescuing mis-splicing in DM1 cell models and the *HSA*^LR^ DM1 mouse model. Importantly, in comparison to the other diamidines, furamidine showed a considerable reduction of off-target effects and toxicity [43,44]. The prodrug of furamidine previously went to phase III clinical trials for African sleeping sickness but was discontinued because of toxicity [45]. However, it is important to note that the dose of furamidine used as a treatment for African sleeping sickness was considerably higher than the equivalent dose necessary to rescue splicing in DM1 patient cell and mouse models [44]. Thus, consideration should be given to furamidine and derivatives of furamidine for clinical trials at lower doses for the treatment of DM. Overall, despite the toxicity issues, the diamidines are a promising class of drugs with therapeutic potential for the treatment of DM. 

### 3.3. Repurposing Drug Screens

Re-purposing existing FDA-approved drugs has tremendous potential to identify therapeutically-relevant compounds that have the potential to get to the clinic faster and with fewer obstacles. A recent targeted screen of 20 FDA-approved antibiotics with RNA interaction potential, identified erythromycin and neomycin (Table S2) [46]. These two compounds displayed the strongest dose-dependent inhibition of the MBNL:r(CUG)_100_ interaction out of the set of 20 antibiotics and were validated using an electrophoretic mobility shift assay (EMSA) [46]. Erythromycin reduced ribonuclear foci and reversed mis-splicing of several events, including *Atp2a1* exon 22 and *Mbnl1* exon 5 and 7 in the C2C12 DM1 transgenic cell model at an optimal dose of 50 µM [46]. Consistent with a mechanism of displacing/blocking the MBNL1:r(CUG)_EXP_ RNA interaction rather than acting on r(CUG)_EXP_ RNA, erythromycin treatment did not significantly alter the levels of r(CUG)_800_ [46]. Treatment with erythromycin also displayed favourable activity in DM1 patient-derived fibroblasts, reducing ribonuclear foci and reversing mis-splicing of various events [46]. Most notably, oral administration of erythromycin in the *HSA*^LR^ DM1 mouse model at the equivalent therapeutic dose currently being used in humans, significantly rescued mis-splicing of several events, including *Clcn1* exon 7a and *Atp2a1* exon 22, and partially rescued myotonia [46]. Importantly, unlike some of the novel compounds identified from other screens, these antibiotics did not display significant toxicity in mice even at doses 2 to 3 times higher than levels used for humans. Taken together, these pre-clinical findings establish a strong foundation for considering erythromycin as a treatment for DM1 in clinical trials, and also highlight drug re-purposing as a powerful therapeutic strategy to identify lead compounds with low toxicity. 

### 3.4. Screening for Disruption of the RNA:RBP Interaction

The interaction between CUG RNA and MBNL is a promising and rich target for small molecule screens. Development of robust high-throughput screens for compounds that disrupt MBNL:r(CUG)n binding in vitro has facilitated the rapid assessment of hundreds of thousands of compounds [47,48]. For example, a fluorescence resonance energy transfer (FRET)-based in vitro MBNL1:r(CUG)_12_ high throughput binding assay screened 279,433 compounds from the National Institutes of Health (NIH) Molecular Libraries Small Molecule Repository (MLSMR) [48]. From this screen, lomofungin, a natural microbial agent derived from *Streptomyces lomondensis,* was identified as the most active compound in inhibiting CUG binding to MBNL1 in vitro. Further investigation showed that this compound also partially rescued mis-splicing of the *Atp2a1* exon 22 Mbnl1-dependent event in a C2C12 DM1 model cell line (Table S2) [48]. Interestingly, lomofungin dimerizes in the presence of DMSO to form dilomofungin, which was even more potent in inhibiting CUG binding to MBNL1 in vitro. Unfortunately, dilomofungin has the undesirable effect of stabilizing the toxic CUG RNAs within cells [48]. To date, the bioactivity of lomofungin or its derivative in a DM1 animal model has not been reported. This study provided proof-of-concept for large-scale screening of compounds disrupting the MBNL:r(CUG)_EXP_ interaction, while also highlighting a limitation to screening compounds solely for high-affinity binding to toxic CUG RNA. 

To identify peptide inhibitors of the toxic CUG RNA process, the Artero group has leveraged the tractability of *Drosophila* as a model for DM to establish an in vivo screening platform [49]. Flies expressing toxic interrupted r(CUG)_480_ RNA in the mushroom bodies of the brain cause a semi-lethal phenotype in female pupae [50]. A peptide mixture library was screened using this *Drosophila* line for suppressors of lethality, identifying the ABP1 peptide (Table S2) [49]. Expression of the ABP1 peptide in transgenic DM1 flies rescued eye and muscle degeneration associated with toxic CUG expression. Interestingly, ABP1 did not competitively displace *Drosophila* Mbl (the fly ortholog of mammalian MBNL/Mbnl) in vitro, supporting distinct binding sites, but it did reduce CUG ribonuclear foci and reversed Mbl sequestration in vivo [49]. The mechanism of action is thought to be due to its ability to shift duplex CUG RNA to a single-stranded form, rather than directly disrupting the Mbl:r(CUG)n interaction. Importantly, treatment of *HSA*^LR^ mice with ABP1 through intramuscular TA injections increased *Clcn1* expression and partially rescued mis-splicing of several events, including *Atp2a1* exon 22, even after 1 month post injection [49]. Although effects on myotonia were not reported in the treated *HSA*^LR^ mice, this approach highlights the potential of in vivo screening and a promising avenue using peptide-based therapy for DM. 

In a more recent screening effort, the Artero group identified daunorubicin as an inhibitor of the MBNL1:r(CUG)_EXP_ interaction in vitro and in DM1 patient-derived myoblasts [51]. Daunorubicin (Table S2) treatment of a *Drosophila* DM1 model expressing 250 pure CUG repeats rescued cardiac dysfunction, including systolic and diastolic dysfunction, arrhythmia, and reduced contractility which was sufficient to increase survival [51]. Taken together with the earlier ABP1 results, these studies highlight the power of *Drosophila* as both a therapeutic screening and validation tool. 

### 3.5. Ribonuclear Foci as a Screening Measure

One of the cellular hallmarks of DM and the toxic RNA:protein sequestration model is the formation of ribonuclear foci composed of CUG/CCUG expansion RNA and MBNL1/2/3 proteins. [52,53,54,55,56,57]. Taking advantage of foci as an important cellular biomarker for DM, small molecule screening has been conducted using fluorescence in situ hybridization (FISH) and imaging to directly monitor changes to the number of ribonuclear foci in DM1/2 patient cell lines [58]. This medium throughput approach was applied to screening several small molecule libraries (>16,000 compounds), leading to the identification of two novel compounds capable of reducing the ribonuclear foci number in both DM1 and DM2 patient cells and partially reverse MBNL-dependent mis-splicing in DM1 patient cells [58]. The first compound, Ro-31-8220 (Table S2), was previously demonstrated to protect against the cardiac conduction and contractile abnormalities in a DM1 heart-specific mouse model through inhibition of PKC-mediated elevation of CUGBP1 (also known as CELF1) protein levels [59]. Ketley et al. demonstrate that Ro-31-8220 functions in a PKC-independent manner to reduce ribonuclear foci. Therefore, Ro-31-8220 appears to exert a dual protective effect in DM by acting to reverse both MBNL and CUGBP1 deregulation, although the precise mechanism and the specific kinase targets have not been fully resolved [58,59]. The second compound, chromomycin A3, is a GC-rich DNA binding agent that can disrupt RNA polymerase activity [60,61]. Because foci number can be affected by either disrupting CUG/CCUG interactions with MBNL proteins or by inhibiting transcription of CUG/CCUG expansion RNAs, chromomycin A3 could potentially work through one or both of these mechanisms. Distinguishing these activities and determining off-target effects is an important next step in considering any utility of this compound for future studies. While the technical difficulties of observing and quantifying RNA foci hampers its effectiveness as a high-throughput screening read-out, RNA foci formation remains a critical biomarker for secondary validation and characterization of therapeutic lead candidates. 

## 4. Upregulating MBNL Protein Levels as a Therapeutic

The direct upregulation of MBNL protein levels is a functional alternative to reducing ribonuclear foci or releasing sequestered MBNL proteins. The MBNL family of proteins (MBNL1, 2, 3) is a compelling target as there is strong evidence their sequestration in particular drive many of the cardinal symptoms of DM [52,53,62,63,64]. While MBNL1 overexpression alone may not be sufficient to rescue all the associated pathogenesis in DM [65], restoring functional levels of MBNL proteins may be a viable therapeutic approach to reverse many of the MBNL-dependent mis-splicing and RNA processing defects driving specific symptoms in DM [66,67]. To this end, a screening strategy was devised to identify small molecules that upregulate MBNL1 protein levels [68]. A clonal screening cell line was engineered by incorporating a ZsGreen fluorescent tag at the N-terminus of endogenous MBNL1 in HeLa cells using the CRISPR/Cas9 system to allow MBNL levels to be monitored using flow cytometry [68]. A targeted pilot screen was conducted using 61 epigenetic modulators on the basis that they may modulate MBNL1 expression at the transcriptional level, leading to increased MBNL1 protein levels [68]. The initial screen identified ISOX and vorinostat (Table S3), both histone deacetylase (HDAC) inhibitors that upregulated MBNL1 levels by ~2 fold at the 2–3 µM range [68]. Treatment of control and DM1 patient-derived fibroblasts with ISOX or vorinostat at 5 µM for 2 days significantly increased MBNL1 levels (up to twofold), which was sufficient to significantly increase the inclusion of the *ATP2A1* exon 22 and *INSR* exon 11 [68]. Subsequent large-scale screening using this assay identified additional hits capable of upregulating MBNL1 levels, several of which were identified from an FDA-approved drug set. While upregulation of MBNL1 levels clearly provides therapeutic benefit in DM, it is important to carefully evaluate the extent of off-target effects of HDAC inhibition on the transcriptome in patient cell and animal models at the appropriate doses. This evaluation, while important for any small molecule screen, is especially important for modulators of transcription. 

Another drug repurposing screen, utilizing nonsteroidal anti-inflammatory drugs (NSAIDs), identified phenylbutazone (PBZ) as a potential compound to alleviate the DM1 pathogenic mechanism (Table S3) [69]. The drug was shown to decrease MBNL binding to the CUG RNA and to increase the transcription of Mbnl1 by suppressing enhancer methylation [69]. Phenylbutazone increased Mbnl1 levels in a C2C12 cell model of DM1 and increased *Mbnl1* mRNA and protein levels in the *HSA*^LR^ DM1 mouse model. In these mice, PBZ treatment increased grip strength, rescued mis-splicing, and reduced the number of central nuclei in the muscle fibers [69]. This work illustrates the advantages of identifying compounds that target multiple steps of CUG RNA toxicity in DM. 

Several of the previously identified compounds may target multiple aspects of the disease pathway. For example, furamidine was recently shown to also upregulate MBNL1 and MBNL2 levels (Table S2) [44]. This finding was surprising, as furamidine had been thought to be involved in binding CTG and CUG repeats and inhibiting transcription and/or affecting RNA stability and the displacement of MBNL proteins. The furamidine-based upregulation of MBNL transcripts and proteins occurs in mouse and human DM models [44], although the basis for this upregulation is currently unknown. These results highlight the need to examine all aspects of the DM disease mechanisms, even for compounds that display the predicted mode of action.

## 5. Leveraging Mis-Splicing as a Read-Out in High-Throughput Screens 

A primary downstream consequence of the toxic RNA gain-of-function model in DM is the spliceopathy associated with MBNL sequestration and hyperphosphorylation of CELF/CUGBP proteins. These downstream events have been directly connected to many aspects of the characteristic pathophysiology observed in DM [52,53,70,71]. Thus, mis-splicing is a very important biomarker in DM reflecting disease severity [72,73], which can be leveraged to monitor the therapeutic potential of small molecules. Numerous small molecule screens have been developed using splicing reporters in cell-based systems and even in vivo [74,75,76]. 

One of the primary symptoms of DM—myotonia—results from mis-splicing of the muscle-specific chloride channel (*CLCN1*) pre-mRNA, leading to a depletion of protein levels [77,78]. Rescue of *CLCN1* mis-splicing is sufficient to improve myotonia [79], and thus can serve as a therapeutically-relevant read-out in small molecule screens. This principle was applied to a high-throughput screen to identify modulators of mis-splicing in DM1 [74]. The system utilized DM1 patient-derived MYOD-inducible, immortalized fibroblasts containing a *CLCN1*-luciferase mini-gene construct to monitor the DM1 *CLCN1* intron 2 retention defect [77,78]. Retention of intron 2 of *CLCN1* in the minigene results in the presence of a premature stop codon and reduced expression of the luciferase reporter [74]. Following MYOD-induced differentiation to DM1 myoblasts, a proof-of-concept screen of ~13,000 compounds was carried out in this system [74]. 

A similar approach was taken by another group, using a mouse *Clcn1* exon 7a luciferase mini-gene reporter construct. Aberrant inclusion of *Clcn1* exon 7a containing a premature stop codon results in reduced luciferase expression in this system [75], such that correction of mis-splicing yields higher luciferase expression [75]. Mouse C2C12 cells were co-transfected with the *Clcn1* exon 7a luciferase reporter construct and a CTG_480_ interrupted construct and were screened for splicing rescue using small molecules from the ICCB Known Bioactives Library. The results identified Ro 31-8220 (Table S2), confirming previous findings [59], but also identifying the antibiotic manumycin A (Table S4) as a novel compound with therapeutic potential in DM1 [75]. The efficacy of the latter drug was confirmed by treating *HSA*^LR^ mice that display aberrant *Clcn1* exon 7a inclusion, with manumycin A via TA injection and demonstrating rescued mis-splicing [75]. While the mechanism of action was determined to be through inhibition of H-Ras consistent with manumycin A functioning as an inhibitor of Ras farnesyltransferase [75], the connection between Ras signaling and mis-splicing was not entirely clear. These results highlight the potential of cell-based mis-splicing screening systems to identify small molecule modulators of important DM mis-splicing events.

Splicing-based screens are not limited simply to cell-based systems, as a splicing reporter system has also been established in a transgenic DM1 *Drosophila* model. This system which facilitates the evaluation of tissue-specific mis-splicing [76] is based upon ‘spliceosensor’ flies that express DM1-relevant mini-genes in frame with a downstream firefly luciferase gene under the control of the UAS-Gal4 system targeted to muscle using a myosin heavy chain (*MHC*-Gal4) driver line [76]. When crossed to flies expressing toxic CUG RNA from an interrupted CTG_480_ construct, luminescence is reduced because of MBNL-dependent mis-splicing of the mini-gene event [76]. One line expressing the *INSR* exon 11 skipping event was selected for an automated high-throughput screen in a 96-well plate format with three larvae per well exposed to the culture media containing the compounds. Larvae were grown for 14 days to allow pupae to develop into adult stages in the presence of compound, following which, flies were frozen, counted, and homogenized, and luminescence was measured to identify hits. Hits that increased luminescence were reflective of a rescue of the *INSR* mis-splicing event [76]. Screening of 16,063 compounds identified several lead compounds that rescued *INSR* mis-splicing, reduced CUG ribonuclear foci, and increased the lifespan of DM1 transgenic flies in subsequent characterizations [76]. It will be interesting to compare these lead candidates versus those from cell-based systems for in vivo properties, including tissue distribution and bioavailability, in DM1 mouse models to evaluate the benefits of screening in vivo. The identification of lead compounds that display good target engagement in affected tissues right from the screening stage has the potential to expedite delivery of promising tissue-relevant therapeutics to the clinic. 

## 6. Restoring CUGBP1 for Therapeutic Benefit in DM1

Hyperphosphorylation and elevation of CUGBP1 protein levels, which is thought to involve the protein kinase C (PKC) pathway [80,81], is another pathogenic hallmark of DM1. There have been several approaches targeting this pathway for therapeutic benefit in DM. The PKC inhibitor Ro-31-8220 (Table S2) has previously been shown to reverse CUGBP1 hyperphosphorylation and upregulation, rescuing some of the associated CUGBP1-dependent splicing defects and improving contractile dysfunction and mortality in a heart-specific DM1 mouse model [59]. In a similar manner, the kinase inhibitors C16 and C51 (Table S4) were also found to stabilize CUGBP1 levels and rescue the associated mis-splicing in DM1 patient-derived fibroblast and myoblast cell lines [82]. In addition to altered protein levels, the activity of CUGBP1 is also deregulated in DM. CUGBP1 is thought to regulate translation of certain mRNAs through interaction with the eukaryotic initiation translation factor 2α (eIF2α) mediated by cyclin D3/CDK4 phosphorylation at S302, and it is this activity which may be disrupted in DM1 [83,84]. DM1 patient muscle biopsy samples where elevated CUGBP1 was observed revealed reduced cyclin D3 levels and increased levels of GSK3β (glycogen synthase kinase 3β, a known negative regulator of cyclin D3) [85]. The same cyclin D3-CUGBP1 expression pattern was also observed in the *HSA*^LR^ mouse model [85] making this model ideal for characterizing CUGBP1-based treatments. In this manner, treatment of *HSA*^LR^ mice with GSK3β inhibitors lithium and 4-benzyl-2-methyl-1,2,4-thiadiazolidine-3,5-dione (TDZD-8) restored the balance of cyclin D3-CUGBP1 (Table S4). This restoration was sufficient to reverse myotonia and grip strength in treated *HSA*^LR^ mice, showing promise for targeting GSK3β as a muscle therapy for DM1 [85]. Most recently, an orally-available GSK3β inhibitor, Tideglusib, was tested in both the *HSA*^LR^ and DMSXL DM1 mouse models with positive effects on survival, growth, and muscle function [86]. Notably, there was also an effect in reducing mutant *DMPK* mRNA levels in both DM1 and congenital myotonic dystrophy patient derived myoblasts [86]. Tideglusib has recently finished a phase II clinical trial for DM1 (clinicaltrials.gov: NCT02858908), although results of the trial have not been officially released. The development of small molecules targeting the CUGBP1 pathway of the DM1 disease mechanism suggests that intervening downstream of the toxic CUG repeats can have significant therapeutic benefit.

## 7. Blocking Transcription of the CTG/CCTG Expansions

There are several elements that make targeting transcription of CTG and CCTG expansions a viable therapeutic strategy in DM. Blocking transcription has the potential to block all the various downstream effects of the toxic RNA, including any potentially unknown or as-of-yet unidentified downstream processes. Furthermore, as there is typically only one expanded allele in DM individuals, small molecules that are specific to the DNA repeat expansion can potentially be administered at lower doses than if targeting the numerous toxic RNA or the MBNL:RNA complexes. These properties make screening and developing small molecules that target transcription an attractive option for DM therapeutics. 

One of the transcription-targeting compounds under investigation is actinomycin D (ActD), a natural compound produced from *Streptomyces* bacteria (Table S5). This drug is currently an FDA-approved anticancer drug used in the clinic under the trade name Cosmegen. ActD functions as a transcription inhibitor by intercalating into GC-rich DNA sequences and blocking progression of the RNA polymerase [87,88,89]. While all three eukaryotic RNA polymerases are sensitive to ActD, early work illuminated a dose-dependent inhibitory effect where genes transcribed by RNA polymerase I are most sensitive, followed by RNA polymerase II and finally III. Both the repetitive nature and the length of the gene sequence being transcribed were predicted to play a role in this inhibitory effect [89]. Subsequent in vitro binding experiments using oligonucleotides supported a strong affinity of ActD for CTG sequences [90]. Structural studies highlighted the importance of the T:T mismatch adjacent to GpC sites as an important determinant for the high-affinity binding of ActD to CTG:CTG DNA substrates [91]. Based on these features, ActD was hypothesized to selectively bind to CTG repeat expansions, block transcription to reduce the toxic CUG RNA load, and rescue the molecular consequences associated with DM1 [92]. Treatment of a DM1 HeLa cell model and patient-derived fibroblasts resulted in a selective reduction of the toxic CUG RNA in the low nanomolar range and a reduction in ribonuclear foci in the HeLa DM1 model (Table S1) [92]. Consistent with the established anticancer activity of ActD, the HeLa cell model displayed some cell toxicity [92]. Treatment of the *HSA*^LR^ DM1 mouse model resulted in a selective reduction of the *HSA* transgene mRNA containing the toxic CUG RNA [92]. Notably, there was modest alteration to the transcriptome with fewer than 4.3% of genes altered at a treatment dose of 0.125 mg/kg [92]. Assessment of Mbnl-dependent mis-splicing in *HSA*^LR^ mice treated at this dose identified the rescue of multiple DM1-relevant events, such as *Clcn1* exon 7a, *Atp2A1* exon 22, and *Mbnl1* exon 5 [92]. While mice treated with ActD at the clinically relevant dose of 0.125 mg/kg did not exhibit signs of general toxicity, the specificity does need to be further refined for a clinically desirable outcome. Overall this work served as proof-of-concept for targeting transcription in a selective manner as a DM therapeutic approach.

Small molecules are not the only method for targeting transcription, as exemplified by recent work by Pinto and colleagues [93]. They showed that deactivated Cas9 (dCas9) with appropriate guide RNAs could target expanded CTG repeats and CCTG repeats and inhibited transcription of RNA in a length-dependent manner [93]. The authors suggested that the impressive reduction of the expanded RNAs was due to the many dCas9-guide complexes coating the longer repeats. Taken together the results from both ActD and dCas9, studies showed selectivity for expanded repeats over short repeats, suggesting that expanded repeats are more susceptible to transcription inhibition compared to short repeats. In this manner, the unique aspect of DM pathogenesis that is the repeat expansion may proof to be the lynchpin to targeting it for therapeutic treatment.

## 8. Targeting Repeat-Associated Non-ATG (RAN) Translation

Repeat-associated non-ATG (RAN) translation is a non-canonical process by which expansion RNA undergo translation in all three reading frames of the repeat tract without the need for a canonical ATG start codon (see recent reviews from the Ranum lab [94,95,96,97]). This process is repeat-tract length and structure-dependent and in combination with bidirectional transcription, and can result in the production of up to six toxic RAN proteins from a single repeat expansion. The accumulation of RAN proteins has been found in the disease-relevant tissue of a growing number of repeat expansion disorders, including spinocerebellar ataxia type 8 (SCA8), Huntington’s disease (HD), C9orf72 ALS/FTD, and myotonic dystrophy type 1 and type 2. While the contribution of individual RAN proteins to disease is an area of active research and an active therapeutic target, RAN translation is also an important factor to consider when designing small molecule strategies to mitigate DM RNA toxicity. Naturally, small molecule approaches that reduce the amount of expansion RNA transcript should theoretically reduce the amount of RNA available for both canonical and non-canonical translation. For example, in DM2, both RNA foci and the nuclear sequestration of expansion CCUG transcripts by MBNL1 were shown to be inversely correlated with sense DM2 LPAC RAN protein expression [98]. It is important to note that DM2 RAN proteins were shown to be toxic independent of RNA gain-of-function [98]. However, any small molecule approaches that free the expansion RNA from MBNL proteins and/or liberates the expansion RNA to the cytoplasm could also result in increased RAN protein production and increased cellular toxicity. Similarly, there is a close connection between RAN translation and cellular stress, such that the activation of the integrated stress response (ISR) pathways can result in increased RAN translation and the accumulation of RAN proteins. In this manner, any off-target effects of small molecule treatments aimed upstream of RAN translation should be considered in the context of the activation of the ISR pathway and the possibility of enhancing RAN translation.

The underlying mechanism(s) of RAN translation is the subject of some debate, yet this pathway represents a promising target for small molecule interventions to reduce RAN translation. This small molecule approach has yielded some promising results, including several bioactive small molecules targeting G_4_C_2_ expansion RNA that significantly inhibit RAN translation and foci formation in cultured cells [99]. The same group later demonstrated that the small molecule, 2H-5-CA-Biotin, improves pre-mRNA splicing defects and selectively inhibits RAN translation in a FXTAS cellular model [100]. Many of these similar approaches target the expansion RNA to reduce RAN translation, rather than directly targeting RAN translation itself. The Disney group recently utilized a cell-based screen for inhibition of RAN translation to identify compounds that selectively inhibit RAN translation from the C9-ALS/FTD G_4_C_2_ expansion [101]. Compound 4 (Table S1) was shown to bind the hairpin structure of the G_4_C_2_ expansion, inhibiting both RNA-binding protein sequestration and the generation of toxic RAN proteins. An alternative approach is to target pathways that upregulate RAN translation, as it was recently found that stress-induced RAN translation upregulation can be reduced by small molecule compounds inhibiting the phospho-eIF2α pathway [102]. Given the close connection between RNA toxicity and the accumulation of toxic RAN proteins, it is paramount to examine both RNA and protein products of repeat expansion in any small molecule screen, regardless of the intended target.

Given that RAN translation has been demonstrated across a wide variety of repeat motifs, and that RAN protein accumulation has been observed in a growing number of expansion disorders including DM1 and DM2, targeting this process is an attractive venture. If the underlying mechanisms of RAN translation is shared between the over 40 repeat expansions disorders, compounds that can modulate this process have the potential to make a broad impact on a large number of patients.

## 9. Modulating DNA Repeat Instability for Therapeutic Benefit in DM

Targeting the expanded DNA repeats, the source of the downstream toxic RNA and protein products for elimination or reduction could be the ideal therapeutic target, but remains a challenging endeavour. There are two basic fundamental approaches: (1) genome editing strategies to delete or remove the repeat, and (2) repeat instability modulators to induce repeat contractions. The difficulty of these approaches is reflected by the lack of current promising lead compounds. The first approach has warranted considerable attention, especially given the recent progress in CRISPR/Cas9 genome editing. Various genome cutters and editors have been used to delete the repeat target with mixed success, with off-target effects being one of the principle concerns [103,104,105,106,107]. While small molecules have been used to target the expanded CUG repeat of DM1 for cleavage [15,31], a similar approach for DNA has yet to be successfully demonstrated. In contrast, there is experimental evidence, in principle, for the second repeat instability modulator approach. Treatment of patient-derived DM1 cell lines with aphidicolin, which inhibits both leading- and lagging-strand synthesis, or emetine, which blocks lagging strand synthesis specifically, significantly enhanced CTG expansions [108]. While this particular approach resulted in small repeat expansions, it is tempting to envision a small molecule that could drive instability in the opposite direction. The identification of a small molecule that induces contractions offers significant advantage over approaches that strive to cut out the entire repeat tract in one fell swoop. Treatment could occur over a long time, especially if the small molecule was effective at sub-clinical doses, allowing for careful monitoring of off-target effects. Screening and identification of small molecule instability drugs can also take advantage of the considerable research into the mechanisms of repeat instability, including the significant role of mismatch repair proteins [109]. The goal of repeat contractions is attractive, especially given the growing complexity of the downstream consequences. Targeting the disease at the true source eliminates the need to consider the role of sense vs. antisense transcription, RNA vs. protein toxicity, and/or the tissue-specific nature of the aforementioned processes. Of particular note is the fact that targeting the expansion process itself rather than specific downstream pathways, may offer therapeutic efficacy across the entire family of repeat expansion disorders.

## 10. The Future Direction of DM Therapeutics

It is clear that DM is a complex disorder with numerous considerations necessary for developing treatments. The majority of therapeutic studies in DM are currently focused on type 1, leaving type 2 in need of more attention. However, based on the overlap in toxic processes and the similar nature of the toxic RNA in both type 1 and 2, many of the existing therapeutic approaches for DM1 may also directly apply to DM2. While these multiple toxic processes enable development of therapies on multiple fronts, it is important to consider how treating one pathogenic aspect of the disease process influences the others. One potential strategy is a combination approach (e.g., different small molecule combinations, small molecules plus ASOs, transcription inhibition plus RAN protein ablation, etc.) targeting multiple processes and allowing synergy in disease modulation. As proof of concept, a recent study reported that two previously characterized compounds that separately displayed efficacy in DM1 models, furamidine and erythromycin, displayed an even greater rescue of mis-splicing in combination than expected from a mere additive effect [110]. Importantly, this combination treatment yielded lower toxicity and fewer off-target effects than when either drug was administered alone in DM1 patient and mouse models [110]. Hence, if combination treatments are a viable therapeutic strategy for treating DM, there are already many unexplored therapeutic avenues that could potentially hold promise. Given the number of small molecule therapies for DM on the horizon, it will be exciting to follow their development. These studies will lay the groundwork for the eventual therapies for treating DM and will likely illuminate disease biology and treatment avenues for other microsatellite expansion disorders involving toxic RNA mechanisms.

## Supplementary Materials

Supplementary materials can be found at https://www.mdpi.com/1422-0067/20/16/4017/s1.

## References

[1] Lin Y., Dent S.Y., Wilson J.H., Wells R.D., Napierala M. (2010). R loops stimulate genetic instability of CTG.CAG repeats. Proc. Natl. Acad. Sci. USA.

[2] Reddy K., Tam M., Bowater R.P., Barber M., Tomlinson M., Nichol Edamura K., Wang Y.H., Pearson C.E. (2011). Determinants of R-loop formation at convergent bidirectionally transcribed trinucleotide repeats. Nucleic Acids Res..

[3] Reddy K., Schmidt M.H., Geist J.M., Thakkar N.P., Panigrahi G.B., Wang Y.H., Pearson C.E. (2014). Processing of double-R-loops in (CAG).(CTG) and C9orf72 (GGGGCC).(GGCCCC) repeats causes instability. Nucleic Acids Res..

[4] Sellier C., Cerro-Herreros E., Blatter M., Freyermuth F., Gaucherot A., Ruffenach F., Sarkar P., Puymirat J., Udd B., Day J.W. (2018). rbFOX1/MBNL1 competition for CCUG RNA repeats binding contributes to myotonic dystrophy type 1/type 2 differences. Nat. Commun..

[5] Udd B., Krahe R. (2012). The myotonic dystrophies: Molecular, clinical, and therapeutic challenges. Lancet Neurol..

[6] Konieczny P., Selma-Soriano E., Rapisarda A.S., Fernandez-Costa J.M., Perez-Alonso M., Artero R. (2017). Myotonic dystrophy: Candidate small molecule therapeutics. Drug Discov. Today.

[7] Lopez-Morato M., Brook J.D., Wojciechowska M. (2018). Small Molecules Which Improve Pathogenesis of Myotonic Dystrophy Type 1. Front. Neurol..

[8] Napierala M., Krzyzosiak W.J. (1997). CUG repeats present in myotonin kinase RNA form metastable “slippery” hairpins. J. Biol. Chem..

[9] Tian B., White R.J., Xia T., Welle S., Turner D.H., Mathews M.B., Thornton C.A. (2000). Expanded CUG repeat RNAs form hairpins that activate the double-stranded RNA-dependent protein kinase PKR. RNA.

[10] Mooers B.H., Logue J.S., Berglund J.A. (2005). The structural basis of myotonic dystrophy from the crystal structure of CUG repeats. Proc. Natl. Acad. Sci. USA.

[11] Childs-Disney J.L., Yildirim I., Park H., Lohman J.R., Guan L., Tran T., Sarkar P., Schatz G.C., Disney M.D. (2014). Structure of the myotonic dystrophy type 2 RNA and designed small molecules that reduce toxicity. ACS Chem. Biol..

[12] Blaszczyk L., Rypniewski W., Kiliszek A. (2017). Structures of RNA repeats associated with neurological diseases. Wiley Interdiscip. Rev. RNA.

[13] Arambula J.F., Ramisetty S.R., Baranger A.M., Zimmerman S.C. (2009). A simple ligand that selectively targets CUG trinucleotide repeats and inhibits MBNL protein binding. Proc. Natl. Acad. Sci. USA.

[14] Wong C.H., Nguyen L., Peh J., Luu L.M., Sanchez J.S., Richardson S.L., Tuccinardi T., Tsoi H., Chan W.Y., Chan H.Y. (2014). Targeting toxic RNAs that cause myotonic dystrophy type 1 (DM1) with a bisamidinium inhibitor. J. Am. Chem. Soc..

[15] Nguyen L., Luu L.M., Peng S., Serrano J.F., Chan H.Y., Zimmerman S.C. (2015). Rationally designed small molecules that target both the DNA and RNA causing myotonic dystrophy type 1. J. Am. Chem. Soc..

[16] Lee J., Bai Y., Chembazhi U.V., Peng S., Yum K., Luu L.M., Hagler L.D., Serrano J.F., Chan H.Y.E., Kalsotra A. (2019). Intrinsically cell-penetrating multivalent and multitargeting ligands for myotonic dystrophy type 1. Proc. Natl. Acad. Sci. USA.

[17] Li J., Matsumoto J., Bai L.P., Murata A., Dohno C., Nakatani K. (2016). A Ligand That Targets CUG Trinucleotide Repeats. Chemistry.

[18] Li J., Nakamori M., Matsumoto J., Murata A., Dohno C., Kiliszek A., Taylor K., Sobczak K., Nakatani K. (2018). A Dimeric 2,9-Diamino-1,10-phenanthroline Derivative Improves Alternative Splicing in Myotonic Dystrophy Type 1 Cell and Mouse Models. Chemistry.

[19] Childs-Disney J.L., Wu M., Pushechnikov A., Aminova O., Disney M.D. (2007). A small molecule microarray platform to select RNA internal loop-ligand interactions. ACS Chem. Biol..

[20] Disney M.D., Childs-Disney J.L. (2007). Using selection to identify and chemical microarray to study the RNA internal loops recognized by 6’-N-acylated kanamycin A. Chembiochem.

[21] Lee M.M., Pushechnikov A., Disney M.D. (2009). Rational and modular design of potent ligands targeting the RNA that causes myotonic dystrophy 2. ACS Chem. Biol..

[22] Lee M.M., Childs-Disney J.L., Pushechnikov A., French J.M., Sobczak K., Thornton C.A., Disney M.D. (2009). Controlling the specificity of modularly assembled small molecules for RNA via ligand module spacing: Targeting the RNAs that cause myotonic muscular dystrophy. J. Am. Chem. Soc..

[23] Wong C.H., Fu Y., Ramisetty S.R., Baranger A.M., Zimmerman S.C. (2011). Selective inhibition of MBNL1-CCUG interaction by small molecules toward potential therapeutic agents for myotonic dystrophy type 2 (DM2). Nucleic Acids Res..

[24] Nguyen L., Lee J., Wong C.H., Zimmerman S.C. (2014). Small molecules that target the toxic RNA in myotonic dystrophy type 2. ChemMedChem.

[25] Disney M.D., Labuda L.P., Paul D.J., Poplawski S.G., Pushechnikov A., Tran T., Velagapudi S.P., Wu M., Childs-Disney J.L. (2008). Two-dimensional combinatorial screening identifies specific aminoglycoside-RNA internal loop partners. J. Am. Chem. Soc..

[26] Pushechnikov A., Lee M.M., Childs-Disney J.L., Sobczak K., French J.M., Thornton C.A., Disney M.D. (2009). Rational design of ligands targeting triplet repeating transcripts that cause RNA dominant disease: Application to myotonic muscular dystrophy type 1 and spinocerebellar ataxia type 3. J. Am. Chem. Soc..

[27] Velagapudi S.P., Seedhouse S.J., French J., Disney M.D. (2011). Defining the RNA internal loops preferred by benzimidazole derivatives via 2D combinatorial screening and computational analysis. J. Am. Chem. Soc..

[28] Childs-Disney J.L., Hoskins J., Rzuczek S.G., Thornton C.A., Disney M.D. (2012). Rationally designed small molecules targeting the RNA that causes myotonic dystrophy type 1 are potently bioactive. ACS Chem. Biol..

[29] Rzuczek S.G., Gao Y., Tang Z.Z., Thornton C.A., Kodadek T., Disney M.D. (2013). Features of modularly assembled compounds that impart bioactivity against an RNA target. ACS Chem. Biol..

[30] Rzuczek S.G., Colgan L.A., Nakai Y., Cameron M.D., Furling D., Yasuda R., Disney M.D. (2017). Precise small-molecule recognition of a toxic CUG RNA repeat expansion. Nat. Chem. Biol..

[31] Angelbello A.J., Rzuczek S.G., McKee K.K., Chen J.L., Olafson H., Cameron M.D., Moss W.N., Wang E.T., Disney M.D. (2019). Precise small-molecule cleavage of an r(CUG) repeat expansion in a myotonic dystrophy mouse model. Proc. Natl. Acad. Sci. USA.

[32] McNaughton B.R., Gareiss P.C., Miller B.L. (2007). Identification of a selective small-molecule ligand for HIV-1 frameshift-inducing stem-loop RNA from an 11,325 member resin bound dynamic combinatorial library. J. Am. Chem. Soc..

[33] Gareiss P.C., Sobczak K., McNaughton B.R., Palde P.B., Thornton C.A., Miller B.L. (2008). Dynamic combinatorial selection of molecules capable of inhibiting the (CUG) repeat RNA-MBNL1 interaction in vitro: Discovery of lead compounds targeting myotonic dystrophy (DM1). J. Am. Chem. Soc..

[34] Ofori L.O., Hoskins J., Nakamori M., Thornton C.A., Miller B.L. (2012). From dynamic combinatorial ‘hit’ to lead: In vitro and in vivo activity of compounds targeting the pathogenic RNAs that cause myotonic dystrophy. Nucleic Acids Res..

[35] Warf M.B., Nakamori M., Matthys C.M., Thornton C.A., Berglund J.A. (2009). Pentamidine reverses the splicing defects associated with myotonic dystrophy. Proc. Natl. Acad. Sci. USA.

[36] Frayha G.J., Smyth J.D., Gobert J.G., Savel J. (1997). The mechanisms of action of antiprotozoal and anthelmintic drugs in man. Gen. Pharm..

[37] Miletti K.E., Leibowitz M.J. (2000). Pentamidine inhibition of group I intron splicing in Candida albicans correlates with growth inhibition. Antimicrob Agents Chemother.

[38] Zhang Y., Bell A., Perlman P.S., Leibowitz M.J. (2000). Pentamidine inhibits mitochondrial intron splicing and translation in Saccharomyces cerevisiae. RNA.

[39] Zhang Y., Li Z., Pilch D.S., Leibowitz M.J. (2002). Pentamidine inhibits catalytic activity of group I intron Ca.LSU by altering RNA folding. Nucleic Acids Res..

[40] Edwards K.J., Jenkins T.C., Neidle S. (1992). Crystal structure of a pentamidine-oligonucleotide complex: Implications for DNA-binding properties. Biochemistry.

[41] Girard R.M., Crispim M., Stolic I., Damasceno F.S., Santos da Silva M., Pral E.M., Elias M.C., Bajic M., Silber A.M. (2016). An Aromatic Diamidine That Targets Kinetoplast DNA, Impairs the Cell Cycle in Trypanosoma cruzi, and Diminishes Trypomastigote Release from Infected Mammalian Host Cells. Antimicrob Agents Chemother.

[42] Coonrod L.A., Nakamori M., Wang W., Carrell S., Hilton C.L., Bodner M.J., Siboni R.B., Docter A.G., Haley M.M., Thornton C.A. (2013). Reducing levels of toxic RNA with small molecules. ACS Chem. Biol..

[43] Siboni R.B., Bodner M.J., Khalifa M.M., Docter A.G., Choi J.Y., Nakamori M., Haley M.M., Berglund J.A. (2015). Biological Efficacy and Toxicity of Diamidines in Myotonic Dystrophy Type 1 Models. J. Med. Chem..

[44] Jenquin J.R., Coonrod L.A., Silverglate Q.A., Pellitier N.A., Hale M.A., Xia G., Nakamori M., Berglund J.A. (2018). Furamidine Rescues Myotonic Dystrophy Type I Associated Mis-Splicing through Multiple Mechanisms. ACS Chem. Biol..

[45] Pohlig G., Bernhard S.C., Blum J., Burri C., Mpanya A., Lubaki J.P., Mpoto A.M., Munungu B.F., N’Tombe P M., Deo G.K. (2016). Efficacy and Safety of Pafuramidine versus Pentamidine Maleate for Treatment of First Stage Sleeping Sickness in a Randomized, Comparator-Controlled, International Phase 3 Clinical Trial. PLoS Negl. Trop. Dis..

[46] Nakamori M., Taylor K., Mochizuki H., Sobczak K., Takahashi M.P. (2016). Oral administration of erythromycin decreases RNA toxicity in myotonic dystrophy. Ann. Clin. Transl. Neurol..

[47] Chen C.Z., Sobczak K., Hoskins J., Southall N., Marugan J.J., Zheng W., Thornton C.A., Austin C.P. (2012). Two high-throughput screening assays for aberrant RNA-protein interactions in myotonic dystrophy type 1. Anal. Bioanal. Chem..

[48] Hoskins J.W., Ofori L.O., Chen C.Z., Kumar A., Sobczak K., Nakamori M., Southall N., Patnaik S., Marugan J.J., Zheng W. (2014). Lomofungin and dilomofungin: Inhibitors of MBNL1-CUG RNA binding with distinct cellular effects. Nucleic Acids Res..

[49] Garcia-Lopez A., Llamusi B., Orzaez M., Perez-Paya E., Artero R.D. (2011). In vivo discovery of a peptide that prevents CUG-RNA hairpin formation and reverses RNA toxicity in myotonic dystrophy models. Proc. Natl. Acad. Sci. USA.

[50] Garcia-Lopez A., Monferrer L., Garcia-Alcover I., Vicente-Crespo M., Alvarez-Abril M.C., Artero R.D. (2008). Genetic and chemical modifiers of a CUG toxicity model in Drosophila. PLoS ONE.

[51] Chakraborty M., Sellier C., Ney M., Pascal V., Charlet-Berguerand N., Artero R., Llamusi B. (2018). Daunorubicin reduces MBNL1 sequestration caused by CUG-repeat expansion and rescues cardiac dysfunctions in a Drosophila model of myotonic dystrophy. Dis. Model. Mech..

[52] Miller J.W., Urbinati C.R., Teng-Umnuay P., Stenberg M.G., Byrne B.J., Thornton C.A., Swanson M.S. (2000). Recruitment of human muscleblind proteins to (CUG)(n) expansions associated with myotonic dystrophy. EMBO J..

[53] Kanadia R.N., Johnstone K.A., Mankodi A., Lungu C., Thornton C.A., Esson D., Timmers A.M., Hauswirth W.W., Swanson M.S. (2003). A muscleblind knockout model for myotonic dystrophy. Science.

[54] Ho T.H., Charlet B.N., Poulos M.G., Singh G., Swanson M.S., Cooper T.A. (2004). Muscleblind proteins regulate alternative splicing. EMBO J..

[55] Mankodi A., Urbinati C.R., Yuan Q.P., Moxley R.T., Sansone V., Krym M., Henderson D., Schalling M., Swanson M.S., Thornton C.A. (2001). Muscleblind localizes to nuclear foci of aberrant RNA in myotonic dystrophy types 1 and 2. Hum. Mol. Genet..

[56] Mankodi A., Teng-Umnuay P., Krym M., Henderson D., Swanson M., Thornton C.A. (2003). Ribonuclear inclusions in skeletal muscle in myotonic dystrophy types 1 and 2. Ann. Neurol..

[57] Fardaei M., Rogers M.T., Thorpe H.M., Larkin K., Hamshere M.G., Harper P.S., Brook J.D. (2002). Three proteins, MBNL, MBLL and MBXL, co-localize in vivo with nuclear foci of expanded-repeat transcripts in DM1 and DM2 cells. Hum. Mol. Genet..

[58] Ketley A., Chen C.Z., Li X., Arya S., Robinson T.E., Granados-Riveron J., Udosen I., Morris G.E., Holt I., Furling D. (2014). High-content screening identifies small molecules that remove nuclear foci, affect MBNL distribution and CELF1 protein levels via a PKC-independent pathway in myotonic dystrophy cell lines. Hum. Mol. Genet..

[59] Wang G.S., Kuyumcu-Martinez M.N., Sarma S., Mathur N., Wehrens X.H., Cooper T.A. (2009). PKC inhibition ameliorates the cardiac phenotype in a mouse model of myotonic dystrophy type 1. J. Clin. Invest..

[60] Berman E., Brown S.C., James T.L., Shafer R.H. (1985). NMR studies of chromomycin A3 interaction with DNA. Biochemistry.

[61] Kaziro Y., Kamiyama M. (1965). Inhibition of Rna Polymerase Reaction by Chromomycin A3. Biochem. Biophys. Res. Commun..

[62] Charizanis K., Lee K.Y., Batra R., Goodwin M., Zhang C., Yuan Y., Shiue L., Cline M., Scotti M.M., Xia G. (2012). Muscleblind-like 2-mediated alternative splicing in the developing brain and dysregulation in myotonic dystrophy. Neuron.

[63] Lee K.Y., Li M., Manchanda M., Batra R., Charizanis K., Mohan A., Warren S.A., Chamberlain C.M., Finn D., Hong H. (2013). Compound loss of muscleblind-like function in myotonic dystrophy. EMBO Mol. Med..

[64] Thomas J.D., Sznajder L.J., Bardhi O., Aslam F.N., Anastasiadis Z.P., Scotti M.M., Nishino I., Nakamori M., Wang E.T., Swanson M.S. (2017). Disrupted prenatal RNA processing and myogenesis in congenital myotonic dystrophy. Genes Dev..

[65] Yadava R.S., Kim Y.K., Mandal M., Mahadevan K., Gladman J.T., Yu Q., Mahadevan M.S. (2019). MBNL1 overexpression is not sufficient to rescue the phenotypes in a mouse model of RNA toxicity. Hum. Mol. Genet..

[66] Kanadia R.N., Shin J., Yuan Y., Beattie S.G., Wheeler T.M., Thornton C.A., Swanson M.S. (2006). Reversal of RNA missplicing and myotonia after muscleblind overexpression in a mouse poly(CUG) model for myotonic dystrophy. Proc. Natl. Acad. Sci. USA.

[67] Chamberlain C.M., Ranum L.P. (2012). Mouse model of muscleblind-like 1 overexpression: Skeletal muscle effects and therapeutic promise. Hum. Mol. Genet..

[68] Zhang F., Bodycombe N.E., Haskell K.M., Sun Y.L., Wang E.T., Morris C.A., Jones L.H., Wood L.D., Pletcher M.T. (2017). A flow cytometry-based screen identifies MBNL1 modulators that rescue splicing defects in myotonic dystrophy type I. Hum. Mol. Genet..

[69] Chen G., Masuda A., Konishi H., Ohkawara B., Ito M., Kinoshita M., Kiyama H., Matsuura T., Ohno K. (2016). Phenylbutazone induces expression of MBNL1 and suppresses formation of MBNL1-CUG RNA foci in a mouse model of myotonic dystrophy. Sci. Rep..

[70] Philips A.V., Timchenko L.T., Cooper T.A. (1998). Disruption of splicing regulated by a CUG-binding protein in myotonic dystrophy. Science.

[71] Timchenko L.T., Miller J.W., Timchenko N.A., DeVore D.R., Datar K.V., Lin L., Roberts R., Caskey C.T., Swanson M.S. (1996). Identification of a (CUG)n triplet repeat RNA-binding protein and its expression in myotonic dystrophy. Nucleic Acids Res..

[72] Nakamori M., Sobczak K., Puwanant A., Welle S., Eichinger K., Pandya S., Dekdebrun J., Heatwole C.R., McDermott M.P., Chen T. (2013). Splicing biomarkers of disease severity in myotonic dystrophy. Ann. Neurol..

[73] Wagner S.D., Struck A.J., Gupta R., Farnsworth D.R., Mahady A.E., Eichinger K., Thornton C.A., Wang E.T., Berglund J.A. (2016). Dose-Dependent Regulation of Alternative Splicing by MBNL Proteins Reveals Biomarkers for Myotonic Dystrophy. PLoS Genet..

[74] O’Leary D.A., Vargas L., Sharif O., Garcia M.E., Sigal Y.J., Chow S.K., Schmedt C., Caldwell J.S., Brinker A., Engels I.H. (2010). HTS-Compatible Patient-Derived Cell-Based Assay to Identify Small Molecule Modulators of Aberrant Splicing in Myotonic Dystrophy Type 1. Curr. Chem. Genom..

[75] Oana K., Oma Y., Suo S., Takahashi M.P., Nishino I., Takeda S., Ishiura S. (2013). Manumycin A corrects aberrant splicing of Clcn1 in myotonic dystrophy type 1 (DM1) mice. Sci. Rep..

[76] Garcia-Alcover I., Colonques-Bellmunt J., Garijo R., Tormo J.R., Artero R., Alvarez-Abril M.C., Lopez Castel A., Perez-Alonso M. (2014). Development of a Drosophila melanogaster spliceosensor system for in vivo high-throughput screening in myotonic dystrophy type 1. Dis. Model. Mech..

[77] Mankodi A., Takahashi M.P., Jiang H., Beck C.L., Bowers W.J., Moxley R.T., Cannon S.C., Thornton C.A. (2002). Expanded CUG repeats trigger aberrant splicing of ClC-1 chloride channel pre-mRNA and hyperexcitability of skeletal muscle in myotonic dystrophy. Mol. Cell.

[78] Charlet B.N., Savkur R.S., Singh G., Philips A.V., Grice E.A., Cooper T.A. (2002). Loss of the muscle-specific chloride channel in type 1 myotonic dystrophy due to misregulated alternative splicing. Mol. Cell.

[79] Wheeler T.M., Lueck J.D., Swanson M.S., Dirksen R.T., Thornton C.A. (2007). Correction of ClC-1 splicing eliminates chloride channelopathy and myotonia in mouse models of myotonic dystrophy. J. Clin. Invest..

[80] Ho T.H., Bundman D., Armstrong D.L., Cooper T.A. (2005). Transgenic mice expressing CUG-BP1 reproduce splicing mis-regulation observed in myotonic dystrophy. Hum. Mol. Genet..

[81] Kuyumcu-Martinez N.M., Wang G.S., Cooper T.A. (2007). Increased steady-state levels of CUGBP1 in myotonic dystrophy 1 are due to PKC-mediated hyperphosphorylation. Mol. Cell.

[82] Wojciechowska M., Taylor K., Sobczak K., Napierala M., Krzyzosiak W.J. (2014). Small molecule kinase inhibitors alleviate different molecular features of myotonic dystrophy type 1. RNA Biol..

[83] Timchenko L.T., Salisbury E., Wang G.L., Nguyen H., Albrecht J.H., Hershey J.W., Timchenko N.A. (2006). Age-specific CUGBP1-eIF2 complex increases translation of CCAAT/enhancer-binding protein beta in old liver. J. Biol. Chem..

[84] Huichalaf C., Sakai K., Jin B., Jones K., Wang G.L., Schoser B., Schneider-Gold C., Sarkar P., Pereira-Smith O.M., Timchenko N. (2010). Expansion of CUG RNA repeats causes stress and inhibition of translation in myotonic dystrophy 1 (DM1) cells. FASEB J..

[85] Jones K., Wei C., Iakova P., Bugiardini E., Schneider-Gold C., Meola G., Woodgett J., Killian J., Timchenko N.A., Timchenko L.T. (2012). GSK3beta mediates muscle pathology in myotonic dystrophy. J. Clin. Invest..

[86] Mei W., Wen-Chin W., Lauren S., Diana L., Ana M., Genevieve G., Nikolai T., Mike S., Lubov T. (2019). Correction of GSK3beta in DM1 reduces the mutant RNA and improves postnatal survival of DMSXL mice. Mol. Cell Biol..

[87] Muller W., Crothers D.M. (1968). Studies of the binding of actinomycin and related compounds to DNA. J. Mol. Biol..

[88] Kamitori S., Takusagawa F. (1992). Crystal structure of the 2:1 complex between d(GAAGCTTC) and the anticancer drug actinomycin D. J. Mol. Biol..

[89] Perry R.P., Kelley D.E. (1970). Inhibition of RNA synthesis by actinomycin D: Characteristic dose-response of different RNA species. J. Cell Physiol..

[90] Chen F.M. (1998). Binding of actinomycin D to DNA oligomers of CXG trinucleotide repeats. Biochemistry.

[91] Hou M.H., Robinson H., Gao Y.G., Wang A.H. (2002). Crystal structure of actinomycin D bound to the CTG triplet repeat sequences linked to neurological diseases. Nucleic Acids Res..

[92] Siboni R.B., Nakamori M., Wagner S.D., Struck A.J., Coonrod L.A., Harriott S.A., Cass D.M., Tanner M.K., Berglund J.A. (2015). Actinomycin D Specifically Reduces Expanded CUG Repeat RNA in Myotonic Dystrophy Models. Cell Rep..

[93] Pinto B.S., Saxena T., Oliveira R., Mendez-Gomez H.R., Cleary J.D., Denes L.T., McConnell O., Arboleda J., Xia G., Swanson M.S. (2017). Impeding Transcription of Expanded Microsatellite Repeats by Deactivated Cas9. Mol. Cell.

[94] Banez-Coronel M., Ranum L.P.W. (2019). Repeat-associated non-AUG (RAN) translation: Insights from pathology. Lab. Invest..

[95] Nguyen L., Cleary J.D., Ranum L.P.W. (2019). Repeat-Associated Non-ATG Translation: Molecular Mechanisms and Contribution to Neurological Disease. Annu. Rev. Neurosci..

[96] Zu T., Pattamatta A., Ranum L.P.W. (2018). Repeat-Associated Non-ATG Translation in Neurological Diseases. Cold Spring Harb. Perspect. Biol..

[97] Cleary J.D., Ranum L.P. (2017). New developments in RAN translation: Insights from multiple diseases. Curr. Opin. Genet. Dev..

[98] Zu T., Cleary J.D., Liu Y., Banez-Coronel M., Bubenik J.L., Ayhan F., Ashizawa T., Xia G., Clark H.B., Yachnis A.T. (2017). RAN Translation Regulated by Muscleblind Proteins in Myotonic Dystrophy Type 2. Neuron.

[99] Su Z., Zhang Y., Gendron T.F., Bauer P.O., Chew J., Yang W.Y., Fostvedt E., Jansen-West K., Belzil V.V., Desaro P. (2014). Discovery of a Biomarker and Lead Small Molecules to Target r(GGGGCC)-Associated Defects in c9FTD/ALS. Neuron.

[100] Yang W.Y., Wilson H.D., Velagapudi S.P., Disney M.D. (2015). Inhibition of Non-ATG Translational Events in Cells via Covalent Small Molecules Targeting RNA. J. Am. Chem. Soc..

[101] Wang Z.F., Ursu A., Childs-Disney J.L., Guertler R., Yang W.Y., Bernat V., Rzuczek S.G., Fuerst R., Zhang Y.J., Gendron T.F. (2019). The Hairpin Form of r(G4C2)(exp) in c9ALS/FTD Is Repeat-Associated Non-ATG Translated and a Target for Bioactive Small Molecules. Cell Chem. Biol..

[102] Cheng W., Wang S., Mestre A.A., Fu C., Makarem A., Xian F., Hayes L.R., Lopez-Gonzalez R., Drenner K., Jiang J. (2018). C9ORF72 GGGGCC repeat-associated non-AUG translation is upregulated by stress through eIF2alpha phosphorylation. Nat. Commun..

[103] Cinesi C., Aeschbach L., Yang B., Dion V. (2016). Contracting CAG/CTG repeats using the CRISPR-Cas9 nickase. Nat. Commun..

[104] van Agtmaal E.L., Andre L.M., Willemse M., Cumming S.A., van Kessel I.D.G., van den Broek W., Gourdon G., Furling D., Mouly V., Monckton D.G. (2017). CRISPR/Cas9-Induced (CTGCAG)n Repeat Instability in the Myotonic Dystrophy Type 1 Locus: Implications for Therapeutic Genome Editing. Mol. Ther..

[105] Provenzano C., Cappella M., Valaperta R., Cardani R., Meola G., Martelli F., Cardinali B., Falcone G. (2017). CRISPR/Cas9-Mediated Deletion of CTG Expansions Recovers Normal Phenotype in Myogenic Cells Derived from Myotonic Dystrophy 1 Patients. Mol. Nucleic Acids.

[106] Dastidar S., Ardui S., Singh K., Majumdar D., Nair N., Fu Y., Reyon D., Samara E., Gerli M.F.M., Klein A.F. (2018). Efficient CRISPR/Cas9-mediated editing of trinucleotide repeat expansion in myotonic dystrophy patient-derived iPS and myogenic cells. Nucleic Acids Res..

[107] Lo Scrudato M., Poulard K., Sourd C., Tome S., Klein A.F., Corre G., Huguet A., Furling D., Gourdon G., Buj-Bello A. (2019). Genome Editing of Expanded CTG Repeats within the Human DMPK Gene Reduces Nuclear RNA Foci in the Muscle of DM1 Mice. Mol. Ther..

[108] Yang Z., Lau R., Marcadier J.L., Chitayat D., Pearson C.E. (2003). Replication inhibitors modulate instability of an expanded trinucleotide repeat at the myotonic dystrophy type 1 disease locus in human cells. Am. J. Hum. Genet..

[109] Schmidt M.H.M., Pearson C.E. (2016). Disease-associated repeat instability and mismatch repair. DNA Repair (Amst).

[110] Jenquin J.R.Y., Yang H., Huigens R.W., Nakamori M., Berglund J.A. (2019). Combination Treatment of Erythromycin and Furamidine Provides Additive and Synergistic Rescue of Mis-splicing in Myotonic Dystrophy Type 1 Models. ACS Pharmacol. Transl. Sci..

