# Supplementary material for: Mitigating RNA Toxicity in Myotonic Dystrophy using Small Molecules
Source: Int J Mol Sci. 2019 Aug 17;20(16):4017. doi: 10.3390/ijms20164017 (PMC6720693; doi:10.3390/ijms20164017)
Supplement: Supplementary file 1 [file ijms-20-04017-s001.pdf]

TABLE 1. Small molecules that target the toxic RNA through rational design.

COMPOUND[1]<br>STRUCTURE[2]	MODEL[3]	ACTIVE<br>CONC.[4]	OBSERVATION[5]
Ligand 1<br>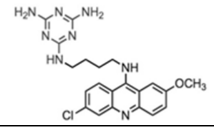	MBNL1:$r(CUG)_{4/12}$	~50uM	CUG-MBNL1 complex disruption
Ligand 3<br>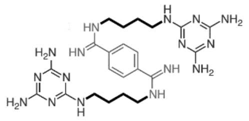	MBNL1:$r(CUG)_{12}$<br>DM1 HeLa cell<br><br>DM1 Drosophila	~10uM<br>50-100uM<br><br>200-400uM	CUG-MBNL1 complex disruption<br>Foci reduction, minigene mis-splicing rescue<br>Rescued a glossy eye phenotype
(New) Ligand 9<br>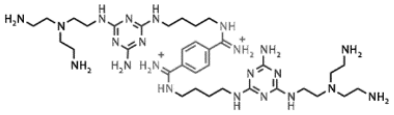	$(CTG{\cdot}CAG)_{74}$<br>$r(CUG)_{16}$<br>DM1 HeLa cell<br><br>DM1 Drosophila	50-100uM<br>5-100uM<br>25-150uM<br><br>100-400uM	Transcription inhibition<br>Cleaved hairpin structure<br>Foci reduction, minigene mis-splicing rescue, reduced $r(CUG)_{exp}$ levels<br>Rescued a glossy eye and larval locomotor phenotypes, reduced $r(CUG)_{exp}$ levels
Ligand 4<br>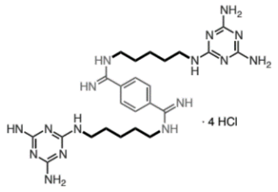	$(CTG{\cdot}CAG)_{74}$<br>$r(CUG)_{16}$<br>DM1 HeLa cell<br><br>DM1 patient-derived cell<br>DM1 Drosophila<br>DM1 liver-specific mouse	0.05-0.4uM<br><br>0.2-4uM<br><br>0.2uM<br>20-80uM<br>2mg/kg	Transcription inhibition<br>Cooperative binding<br>Foci reduction, minigene mis-splicing rescue, reduced $r(CUG)_{exp}$ levels<br>Foci reduction<br>Rescued a progressive climbing defect<br>Foci reduction, mis-splicing rescue, reduced $r(CUG)_{exp}$ levels
DAP<br>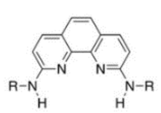	$r(CUG)_9$ or $r(CAG)_9$<br>$d(CUG)_{20}$ luciferase reporter	25uM	Higher binding affinity for $r(CUG)_9$<br>Decrease luciferase expression
DDAP<br>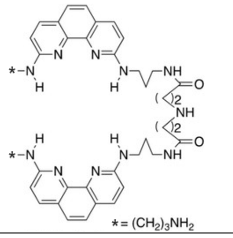	$r(CUG)_9$ or $r(CAG)_9$<br>MBNL1:$r(CUG)_{20}$<br>DM1 C2C12 cell<br>DM1 HSA[LR] mouse	30-120nM<br>10-200nM<br>10-40uM<br>20 or 50mg/kg	Higher binding affinity for $r(CUG)_9$<br>CUG-MBNL1 complex disruption<br>Mis-splicing rescue<br>Mis-splicing rescue
2K-4 and 3K-4<br>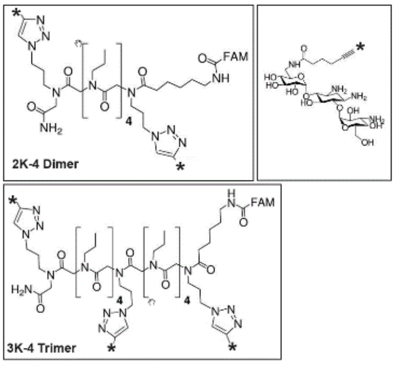	MBNL1:$r(CCUG)_{24}$<br>DM2 C2C12 cell	8-50nM<br>5-20uM	CCUG-MBNL1 complex disruption<br>Minigene mis-splicing rescue

Ligands 6, 8, 10, 11 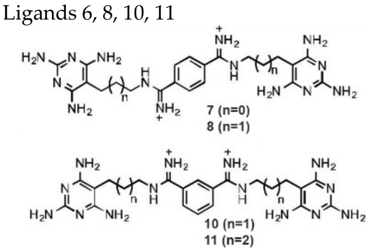	MBNL1:r(CCUG)$_8$<br>DM2 HeLa cell	15-75uM<br>100uM	CCUG-MBNL1 complex disruption<br>Foci reduction
Bis-benzimidazole 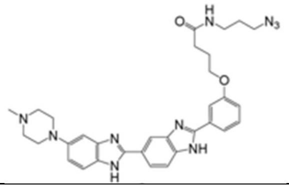	MBNL1:r(CUG)$_{109}$	110uM	CUG-MBNL1 complex disruption
2H-4 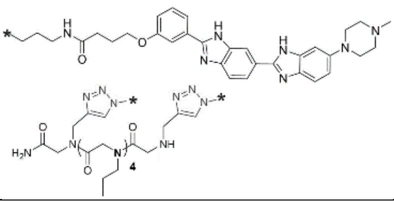	MBNL1:r(CUG)$_{109}$<br>DM1 HeLa cell	11uM<br>5-25uM	CUG-MBNL1 complex disruption<br>Minigene mis-splicing rescue, foci reduction
2H-K4NMeS 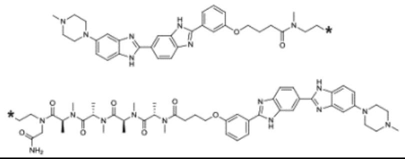	MBNL1:r(CUG)$_{12}$<br>DM1 patient-derived cell	 10nM	CUG-MBNL1 complex disruption<br>Foci reduction, mis-splicing rescue
Cugamycin 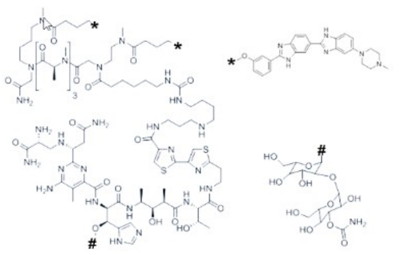	DM1 patient-derived cell<br>DM1 HSA^{LR} mouse	1-4uM<br>10mg/kg	Foci reduction, mis-splicing rescue<br>Foci reduction, mis-splicing rescue, myotonia rescue

[1] Name of compound.
[2] Chemical structure of compound.
[3] *In vitro* or *in vivo* model used.
[4] Concentrations/ranges or doses where effect was observed in DM model (micromolar concentrations or mg/kg of body weight doses).
[5] Observations of compound effect on DM molecular pathogenesis or phenotypes in DM model used.

TABLE 2. Small molecules identified via screens that target toxic CUG RNA.

COMPOUND[1] STRUCTURE[2]	MODEL[3]	ACTIVE CONC.[4]	OBSERVATION[5]
Compound 4	MBNL1:$r(CUG)_{109}$	1-100uM	CUG-MBNL1 complex disruption
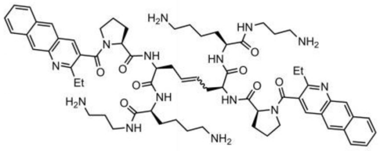	DM1 HSA^{LR} mouse	40mg/kg	Mis-splicing rescue
Compound 11	MBNL1:$r(CUG)_{109}$	1-100uM	CUG-MBNL1 complex disruption
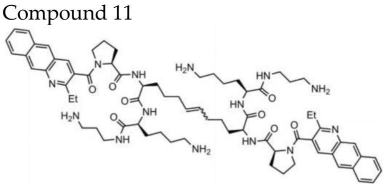	DM1 HSA^{LR} mouse	40mg/kg	Mis-splicing rescue
Pentamidine 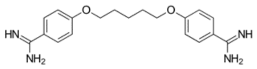	DM1 HeLa cell	10-75uM	Foci reduction, minigene mis-splicing rescue, reduced $r(CUG)_{exp}$ levels
	$(CTG{\cdot}CAG)_{54}$	10-100uM	Transcription inhibition
	DM1 HSA^{LR} mouse	25-40mg/kg	Mis-splicing rescue
Heptamidine	DM1 HeLa cell	5-15uM	Minigene mis-splicing rescue
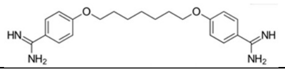	DM1 HSA^{LR} mouse	25-40mg/kg	Mis-splicing rescue, reduced $r(CUG)_{exp}$ levels, myotonia rescue
Furamidine	MBNL1:$r(CUG)_{4}$	10-125uM	CUG-MBNL1 complex disruption
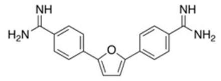	DM1 HeLa cell	20-80uM	Foci reduction, mis-splicing rescue
	DM1 patient-derived cell	0.25-4uM	Foci reduction, mis-splicing rescue, rescued gene expression, increase MBNL1/2 protein levels
	DM1 HSA^{LR} mouse	10-30mg/kg	Mis-splicing rescue, rescued gene expression, reduced $r(CUG)_{exp}$ levels
Neomycin 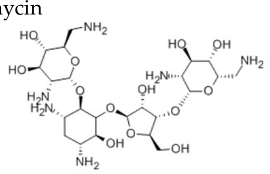	MBNL1:$r(CUG)_{100}$	4-64uM	CUG-MBNL1 complex disruption
Erythromycin	MBNL1:$r(CUG)_{100}$	4-64uM	CUG-MBNL1 complex disruption
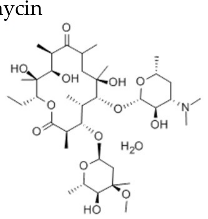	DM1 C2C12 cell	50uM	Foci reduction, mis-splicing rescue
	DM1 patient-derived cell	25-100uM	Foci reduction, mis-splicing rescue
	DM1 HSA^{LR} mouse	150-900mg/kg	Mis-splicing rescue, myotonia rescue
Lomofungin	MBNL1:$r(CUG)_{12}$	0.01-1uM	CUG-MBNL1 complex disruption
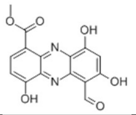	DM1 fluc800 cell	10uM	Increased nucleoplasmic MBNL1 levels
	DM1 C2C12 cell	10uM	Mis-splicing rescue
Ro 31-8220	DM1 patient-derived cell	5-10uM	Foci reduction, mis-splicing rescue, downregulation of CELF1 levels, increased cytoplasmic levels of MBNL
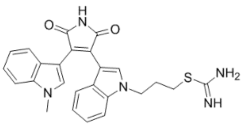	DM2 patient-derived cell	5-10uM	Foci reduction, increased cytoplasmic levels of MBNL
	DM1 Zebrafish	5uM	Body length phenotype rescue

	DM1 heart-specific mouse	6mg/kg	Improved cardiac conduction and contractile abnormalities, mis-splicing rescue
APB1 peptide<br>Ac-ppyawe-NH_2*	DM1 Drosophila	40-250uM	Increased muscle area, rescued eye and muscle degeneration, foci reduction
	DM1 HSA^{LR} mouse	0.5 or 10ug	Mis-splicing rescue, increased Clcn1 expression, reduced central nuclei
Daunorubicin<br>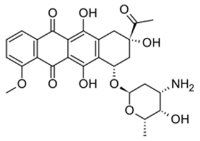	MBNL1:$r(CUG)_{26}$	10uM	CUG-MBNL1 complex disruption
	DM1 patient-derived cell	3-10uM	Foci reduction
	DM1 Drosophila	1uM	Rescued cardiac dysfunction, increased survival, foci reduction, mis-splicing rescue
Chromomycin A3<br>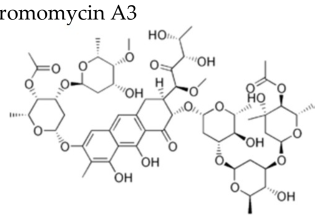	DM1 patient-derived cell	0.04-4uM	Foci reduction, mis-splicing rescue, downregulation of CELF1 levels, increased cytoplasmic levels of MBNL
	DM1 patient-derived cell	0.04-4uM	Foci reduction, increased cytoplasmic levels of MBNL

[1] Name of compound.
[2] Chemical structure of compound.
[3] *In vitro* or *in vivo* model used.
[4] Concentrations/ranges or doses where effect was observed in DM model (micromolar concentrations or mg/kg of body weight doses).
[5] Observations of compound effect on DM molecular pathogenesis or phenotypes in DM model used.
*Represents the lower case one-letter code for D-amino acids.

TABLE 3. Small molecules that upregulate MBNL protein levels.

COMPOUND[1]<br>STRUCTURE[2]	MODEL[3]	ACTIVE<br>CONC.[4]	OBSERVATION[5]
ISOX<br>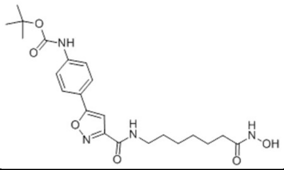	MBNL-tag HeLa cell<br>DM1 patient-derived cell	1-6uM<br>5uM	Increased MBNL1 protein levels<br>Increased MBNL1 protein levels, mis-splicing rescue
Vorinostat<br>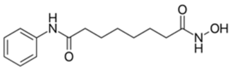	MBNL-tag HeLa cell<br>DM1 patient-derived cell	1-6uM<br>5uM	Increased MBNL1 protein levels<br>Increased MBNL1 protein levels, mis-splicing rescue
Phenylbutazone (PBZ)<br>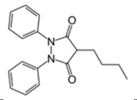	C2C12 cell<br>DM1 HSA^{LR} mouse	50-950uM<br>16.7mg/kg	Increased *Mbnl1* expression<br>Increased Mbnl1 protein levels, mis-splicing rescue, improved grip strength, reduction of central nuclei

[1] Name of compound.
[2] Chemical structure of compound.
[3] *In vitro* or *in vivo* model used.
[4] Concentrations/ranges or doses where effect was observed in DM model (micromolar concentrations or mg/kg of body weight doses).
[5] Observations of compound effect on DM molecular pathogenesis or phenotypes in DM model used.

TABLE 4. Small molecules that affect downstream signaling pathways or restore CUGBP1 activity.

COMPOUND[1]<br>STRUCTURE[2]	MODEL[3]	ACTIVE<br>CONC.[4]	OBSERVATION[5]
Manumycin A<br>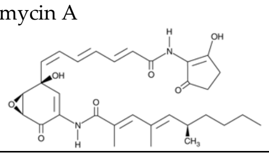	DM1 C2C12 cell<br>DM1 HSA^{LR} mouse	10-40uM	Mis-splicing rescue<br>Mis-splicing rescue
Lithium<br>Li^{+}	DM1 HSA^{LR} mouse	0.24% diet	Improved grip strength, reduced central nuclei, myotonia rescue
TDZD-8<br>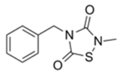	DM1 HSA^{LR} mouse	10mg/kg	Improved grip strength, myotonia rescue
Tideglusib<br>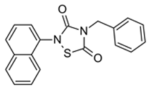	Phase II clinical trials	400mg, 1000mg	Safety and efficiency
Actinomycin D<br>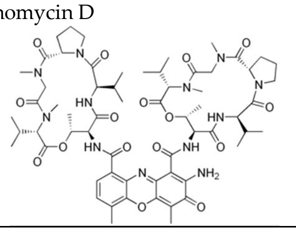	DM1 HeLa cell<br><br>DM1 patient-derived cell<br>DM1 HSA^{LR} mouse	0.005-0.02uM<br><br>5-10nM<br>1.25-2.5mg/kg	Foci reduction, reduction of $r(CUG)_{exp}$ levels<br>Reduced $r(CUG)_{exp}$ levels<br>Mis-splicing rescue
C-16<br>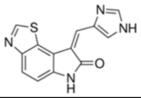	DM1 patient-derived cell	1uM	Downregulation of CELF1, foci reduction, mis-splicing rescue
C-51<br>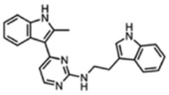	DM1 patient-derived cell	30uM	Downregulation of CELF1, foci reduction, mis-splicing rescue

[1] Name of compound.
[2] Chemical structure of compound.
[3] *In vitro* or *in vivo* model used.
[4] Concentrations/ranges or doses where effect was observed in DM model (micromolar concentrations or mg/kg of body weight doses).
[5] Observations of compound effect on DM molecular pathogenesis or phenotypes in DM model used.

TABLE 5. Small molecules that block transcription of the CTG expansion.

COMPOUND[1] STRUCTURE[2]	MODEL[3]	ACTIVE CONC.[4]	OBSERVATION[5]
Actinomycin D 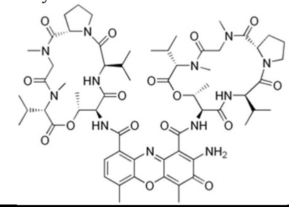	DM1 HeLa cell	0.005-0.02uM	Foci reduction, reduction of $r(CUG)_{exp}$ levels
	DM1 patient-derived cell	5-10nM	Reduced $r(CUG)_{exp}$ levels
	DM1 HSA^{LR} mouse	1.25-2.5mg/kg	Mis-splicing rescue

[1] Name of compound.
[2] Chemical structure of compound.
[3] *In vitro* or *in vivo* model used.
[4] Concentrations/ranges or doses where effect was observed in DM model (micromolar concentrations or mg/kg of body weight doses).
[5] Observations of compound effect on DM molecular pathogenesis or phenotypes in DM model used.
